# Dynamical methods for evaluating the time-dependent unfolding of social coordination in children with autism

**DOI:** 10.3389/fnint.2013.00021

**Published:** 2013-04-08

**Authors:** Paula Fitzpatrick, Rachel Diorio, Michael J. Richardson, R. C. Schmidt

**Affiliations:** ^1^Psychology Department, Assumption CollegeWorcester, MA, USA; ^2^Psychology Department, College of the Holy CrossWorcester, MA, USA; ^3^Psychology Department, University of CincinnatiCincinnati, OH, USA

**Keywords:** autism spectrum disorders, dynamics, social coordination, social competence, time series analyses

## Abstract

Children with Autism Spectrum Disorder (ASD) suffer from numerous impairments in social interaction that affect both their mental and bodily coordination with others. We explored here whether interpersonal motor coordination may be an important key for understanding the profound social problems of children with ASD. We employed a set of experimental techniques to evaluate not only traditional cognitive measures of social competence but also the dynamical structure of social coordination by using dynamical measures of social motor coordination and analyzing the time series records of behavior. Preliminary findings suggest that children with ASD were equivalent to typically developing children on many social performance outcome measures. However, significant relationships were found between cognitive social measures (e.g., intentionality) and dynamical social motor measures. In addition, we found that more perceptually-based measures of social coordination were not associated with social motor coordination. These findings suggest that social coordination may not be a unitary construct and point to the promise of this multi-method and process-oriented approach to analyzing social coordination as an important pathway for understanding ASD-specific social deficits.

Children with Autism Spectrum Disorder (ASD) exhibit numerous impairments in social interaction that typically persist throughout adolescence and adulthood (American Psychiatric Association, 2000; Howlin et al., 2004). These deficits severely impede mental and physical development, learning, and behavioral functioning at home and in the community and also make successful treatment difficult. The processes underlying these impairments are not yet fully understood but seem to affect both their mental and bodily coordination with others. Social interaction involves (a) coordinating thoughts and ideas to establish and maintain a mental connection with another person (e.g., social mental connection); and (b) movement coordination of one's body with another person while performing actions (social motor coordination).

Past research has found that the lack of social competence of children with ASD is comprised of deficits in a number of componential areas including social cognitive (Baron-Cohen, 1995) and social perceptual processes (Klin et al., 2002). Interacting competently with others relies on making inferences about another's mental state and goals (Baron-Cohen and Swettenham, 1997), being able to recognize emotion in various affective expressions (Bauminger, 2002), and understanding the social contextual meaning of those expressions for social interactions (Happe and Frith, 2006).

In addition, a less obvious component of social competence lies within social motor processes, the interpersonal coordination of movements during a social interaction. Indeed, social psychological research has found that social motor coordination both in the form of imitation and in the lesser known phenomenon of interactional synchrony, is important for maintaining critical aspects of successful human social interaction, including interpersonal responsiveness, social rapport and other-directedness (Bernieri et al., 1994; Lakin and Chartrand, 2003), positive self-other relations (Miles et al., 2009; Seger and Smith, 2009), and verbal communication and comprehension (Semin, 2007; Shockley et al., 2009). Past research has also found that breakdowns in social motor coordination are associated with psychological dysfunction such as schizophrenia (Ramseyer and Tschachter, 2011; Varlet et al., 2012) and borderline personality disorders (Gratier and Apter-Danon, 2008) as well as marital dissatisfaction (Julien et al., 2000). Dowd et al. (2010) have recently proposed that understanding motor impairments in autism is important because motor impairments happen in parallel with social and behavioral deficits, may contribute to the social deficits, and may share similar neural circuits.

In fact, motorically-based connections to others such as imitation seems to play an important role in the development and maintenance of social interactions (e.g., Piaget, 1951/1967; Trevarthen, 1998; Meltzoff, 2005). Synchronized bodily coordination has also been proposed to be a basis for the development of intersubjectivity in that it provides a basis for “sharing time” and has also been proposed to be predictive of later more cognitive developmental social outcomes, such as attachment and empathy (Feldman, 2007).

Whereas both imitation and interactional synchrony are evident shortly after birth, more cognitive forms of social connectedness emerge later. Joint Attention emerges around 9 months, develops more fully during second year of life (Tomasello, 1999; Mundy and Newell, 2007; Mundy, 2009), and has been found to be related to individual differences in the emergence of social competence later in childhood (Vaughan Van Hecke et al., 2007). The ability to understand the thoughts and beliefs of others or have a theory of mind develops later still between the second and fourth year of life. Whereas verbal theory of mind tasks suggest that theory of mind develops after 4-years-of age (e.g., Wellman et al., 2001), non-verbal theory of mind tasks and tasks that demonstrate emulation of unfulfilled goals suggest that theory of mind begins to emerge much earlier (Meltzoff, 1995; Woodward, 1998; Carpenter et al., 2001, 2002; Onishi and Baillargeon, 2005). In fact, more complex cooperation tasks that require understanding the goal of another, sharing the goal, and coordinating actions are evident in typically developing children between 18 and 24 months (Warneken et al., 2006).

Due to the fact that these more cognitive aspects of social competence are known to be impaired in children with ASD and that imitation abilities appear to be of foundational importance in the development of such skills, much research has explored the imitative abilities of children with ASD. Indeed, some researchers have proposed that understanding early deficits in the ability to imitate others, along with the possible role of an atypically functioning mirror neuron system, are key to understanding the more cognitive aspects of social deficits in ASD (Rogers and Pennington, 1991; Charman et al., 1997; Williams et al., 2001; Rogers et al., 2003; Gallese, 2006; Oberman and Ramachandran, 2007; Colombi et al., 2009; Rizzolatti and Fabbri-Destro, 2010). Other research, however, suggests that some children with ASD do not have deficits in imitative movements and that the mirror neuron system of the social brain may not be damaged (Hamilton et al., 2007; Gowen et al., 2008; Fan et al., 2010). The lack of consensus with regards to impairments in imitation is perhaps due to methodological differences, including variability in task difficulty and participant characteristics.

Additionally, past research has also shown that children with ASD have profound deficits in the later more cognitive aspects of social competence; however, this research too is sometimes contradictory. For example, children with ASD have been found to have profound deficits in initiating joint attention, but deficits in responding to joint attention seem dependent on mental age–those with lower mental age have deficits in responding, but those with higher mental age do not (Mundy, 2009). Further, while children with ASD perform poorly on verbal theory of mind tasks (Baron-Cohen et al., 1985; Reed, 1994; Hamilton et al., 2007), they have been found to be equivalent to typically developing children in emulating the intended actions of others (Carpenter et al., 2001) and in helping tasks (Liebal et al., 2008). This unexpected finding could mean that children with ASD actually do understand the intentional states of others, but that apparent deficits in joint attention and theory of mind are a consequence of other processes, such as motor control problems (Gernsbacher et al., 2008) or language problems. Similarly, Leekam et al. (1997) have suggested that poor joint attention skills may be due to difficulties making self-generated, spontaneous responses. They attributed this to a lack of social motivation but it is unclear whether or not an underlying motor control problem is the core deficit. Finally, the finding that children with ASD had poorer social competence on complex cooperation tasks (Liebal et al., 2008; Colombi et al., 2009) raises questions about whether the nature of the social deficits are a result of an inability to share goals or coordinate complex action sequences.

Contradictory findings and unexpected social competencies in some tasks make it difficult to develop a comprehensive understanding of the social competencies and social deficits of children with ASD. We maintain that past research's conceptual focus on imitation and mirror neurons and methodological use of behavioral coding measures may not have been nuanced enough to capture the multiple dimensions of the social competence deficits in children with ASD. Theoretical advances in embodied cognition (Chartrand and Bargh, 1999; Dale and Spivey, 2006; Knoblich and Sebanz, 2006; Semin and Cacioppo, 2008; Semin and Smith, 2008; Smith, 2008; Richardson et al., 2010) suggest that if cognitive processes are embodied in social interactions, we should expect to see the social mental connection of individuals reflected in the coordinated states of their bodies (e.g., social motor coordination). Fortunately, recent advances in the dynamics of motor coordination have provided new methods and models for investigating and understanding social motor coordination processes (Schmidt and Richardson, 2008; Schmidt et al., 2011). These techniques allow one to evaluate the dynamical structure of social coordination by using process-oriented measures of social coordination and analyzing the time series records of the time-dependent unfolding of social coordination during social interaction tasks. To evaluate the interaction in time, a recently developed video-based analysis method (Ramseyer and Tschachter, 2011; Schmidt et al., 2012; Paxton and Dale, in press) provides a measure of body movements. Traditional linear (e.g., relative phase, cross-correlation) dynamical time-series techniques allow the evaluation of the patterning and stability of coordination in space-time.

Given all the inconsistency in the literature and the fact that less research has explored the synchronized movement deficits in ASD even though findings indicate that, like imitation, the ability to move in synchrony with another seems to be impaired early and may consequently impact the development of intersubjectivity (Trevarthen and Daniel, 2005; Yirmiya et al., 2006), this paper evaluates the usefulness of the dynamical techniques for exploring the relationship between motorically-based and cognitively-based conceptions of social competence. We suggest that the question of *whether* children with ASD are able to demonstrate a skill may be a less important question than *how* they execute the behavior. If an important dimension of our social connection to others is embodied in the way we move with respect to other people, then an impairment in motor coordination could result in a breakdown in social connection even if a task is “successfully” accomplished. In addition, if *how* is the important question, the critical behavioral measure is not *whether* a task is accomplished but *how* the behavior unfolds over time. As a result, we employed a set of experimental techniques to evaluate not only traditional cognitive measures of social competence but also the dynamical structure of social coordination by using unique, process-oriented measures of social coordination and analyzing the time series records of the time-dependent unfolding of social coordination during social interaction tasks. In particular, we explored how the cognitive or mental measures of coordination correspond to the social motor measures. We expect that participants with ASD will demonstrate deficits in social motor coordination compared to typically developing (TD) participants. Further, based upon past research in normal adults that has found social measures such as rapport and cooperation are related to motor measures of interactional synchrony and imitation, we expect that perceptually-based measures of social competence (joint attention) will be correlated with social motor coordination but more conceptually-based measures of social competence (understanding of intentionality) will not. Finally, we expect that in spite of the fact that overall task success may be similar, a finer-grained dynamical analysis will show that children with ASD were less socially coordinated with the experimenter than TD.

## Method

### Participants

Eighteen children participated in the study and comprised two groups: autism spectrum disorder (ASD, *n* = 11, 5 completed the synchrony task, 6 the imitation task) and typically developing children (TD, *n* = 7, 3 completed the synchrony task, 4 the imitation task). Children with ASD were recruited through advertisements at autism support groups for families with children with autism and local therapist offices, and the TD children were recruited from local preschools. The mean age of children with autism was 76.4 months (Range 59–89 months) and the mean age of the typically developing children was 70.29 months (Range 49–94 months), *t*_(16)_ = 0.92, *p* > 0.05. There were 10 males and 1 female in the ASD group and 4 males and 3 females in the TD group. Parental report of a diagnosis of ASD was used for inclusion in the ASD group. Parents reported that their child had received neuropsychological testing by a clinical psychologist (using either DSM-IV criteria and/or the Autism Diagnostic Observation Schedule, ADOS) and reported the date of the diagnosis. ADOS scores were not recorded. Each participant was given a $10 gift card for his/her participation in the study. The research project was approved by the IRB at Assumption College and College of the Holy Cross. Parents signed an informed consent form and verbal assent was received from the children.

### Cognitive social coordination tests

Paper and pencil parental reports of basic skills and behaviors were completed to assess general development. In addition, tests were performed to evaluate the participants' cognitive social coordination abilities of joint attention, understanding other minds and understanding intentionality. Tests were also performed to test participant's social knowledge more realistically in tasks that required helping others or cooperating with others. These measures are described below.

#### Developmental profile III

The parents of all participants completed the Developmental Profile III (Alpern, 2007), an instrument that screens for developmental delays. It provides scores on five different areas of development: physical, adaptive behavior, social-emotional, cognitive, and communication.

#### Joint attention tasks

Two measures from the Early Social Communication Scales (ESCS: Mundy et al., 2003) were adapted to measure responding to joint attention (RJA) and initiating joint attention (IJA). Even though the ESCS was developed for children between the ages of 8–30 months, the RJA and IJA tasks are very similar to the gaze monitoring tasks and eye contact in ambiguous situations (Leekam et al., 1997; Warreyn et al., 2005) that have been used with older children and the ESCS has well-established coding guidelines. The Gaze Following Task was used to measure RJA. In this task, a poster was positioned to the left of the child, behind the child and to the left, to right of the child, and behind the child to the right. After calling the child's name, the experimenter looked and pointed to each of the four posters in the order that they were listed above. The Gaze Following Task was repeated twice during the experimental session. Experimenters measured RJA by calculating the percent of responses in which the child orients to the poster.

The Object Spectacle Task adapted from the ESCS was used to measure initiating joint attention (IJA). This task was repeated three times during the experimental session using a different toy (2 wind-up mechanical toys and 1 hand-held mechanical toy) for each trial. During each trial, the experimenter activated the wind-up toy or played with the mechanical toy for approximately 6 s. If the child initiated a bid (e.g., making eye contact between the object and tester), the experimenter responded with a brief acknowledgement of the child's request (e.g., smiling and nodding). If the child reached to obtain or asked for the toy, the experimenter put the object within reach of the child. However, if the child made no bid to obtain the object during the 6 s, the experimenter placed the object within reach of the child. After the child was given approximately 10 s to play with the toy, the experimenter retrieved the toy and repeated the task two more times. Experimenters obtained a total score for IJA following the coding guidelines outlined in the ESCS (Mundy et al., 2003).

#### Theory of mind task

A task similar to the Sally-Anne task developed by Baron-Cohen et al. (1985) was used to examine a child's theory of mind or the ability to understand that what another person knows may be different from what he/she knows. The experimenter performed a skit for the child using two small dolls of Gabriela and Gerald, characters from the television series *Sid the Science Kid*. In the skit, Gabriela places a marble in a small box and then goes outside to play. Sid takes the marble from the box and places it in his small, white bag. When Gabriela comes back inside, the experimenter asked the child a series of three questions: “Where will Gabriela look for the marble?,” “Where is the marble really?,” and “Where was the marble to begin with?.” The experimenter coded whether the child answered the questions correctly.

#### Intentionality tasks

To evaluate the child's ability to understand the goals of another, a series intentionality tasks similar to those of Meltzoff (1995) were used. During these tasks, the experimenter demonstrated an action three times on the four different objects. However, during each presentation, the experimenter unsuccessfully completed the intended action. For example, the first object was a dumbbell-shaped toy that could be pulled apart and put back together. During the demonstration, the experimenter tried but failed to pull the dumbbell apart. The second object was a prong and loop toy. During the demonstration, the experimenter tried but failed to hang the loop on the prong. The third object was a square and post toy was made from a transparent plastic square and a wooden dowel. During the demonstration, the experimenter tried but failed to fit the plastic square over the opening of the dowel. The fourth object was a cylinder and beads toy. During the demonstration, the experimenter tried but failed to drop the loop of beads in the metal can. The child did not receive any points for playing with the toy in a way that was unrelated to the actions that the experimenter performed or the intended action. The child received one point if he/she mimicked the experimenter's action. The child received two points if he/she completed the intended action.

#### Helping and cooperation tasks

Helping tasks used were those employed by Liebal et al. (2008). The first task tested whether the participant helped the experimenter pick up a dropped pen. The pen was dropped within reach of the child. During the paper balls task, a box half filled with paper balls was placed in front of the experimenter. The experimenter used tongs to place other paper balls in the box. The test was whether the participant would help the experimenter to reach the two paper balls out of reach. In the clothespins task, the experimenter used clothespins to hang two infant socks on a line that ran from one side of the table to the other. Here the test was whether the participant would help the experimenter when she “accidently” dropped a clothespin to the floor and was unable to reach it. The number of times the child helped in the three tasks was recorded.

The first cooperation task was the double-tube task from Warneken et al. (2006). During this task, a double tube apparatus, consisting of one blue tube and one while tube, was placed on the table. To demonstrate the task, the experimenter dropped a block into the blue tube. A second experimenter was at the lower end of the tubes and positioned a cup underneath the blue tube to catch the block. The experimenter repeated this procedure two more times dropping the block down the white tube. The test was whether the participant would cooperate with the experimenter to play both the roles of letting the block go and catching it. During this task, an interruption period was employed once when the participant was in the role of dropping the block and once when the participant was in the role of catching the block. During the interruption period the experimenter had a neutral expression and avoided making eye contact with the participant for 10 s. After the 10 s passed, the experimenter resumed playing the game. The experimenter coded for whether the child successfully caught the wooden block in the cup. The child's behavior during the interruption period was also coded. The experimenter coded the child's overall behavior as either disengaged or orientated towards the experimenter.

**Figure 1:**
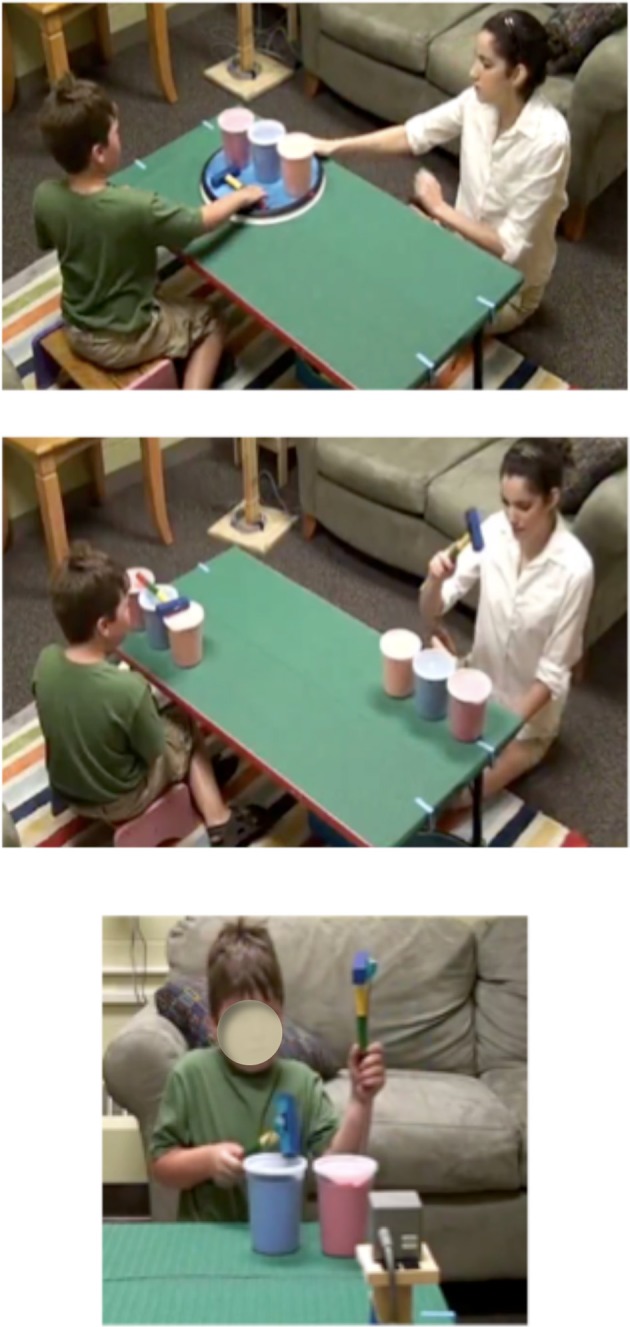
**Illustrations of the experimental set-up.** The top panel displays the turn-taking cooperation task, the middle panel the imitation and synchrony tasks, and the bottom panel the drumming task.

The second cooperation task was a turn-taking task developed by the experimenters as a measure of cooperation. In this task, three different colored cylinders were placed in a horizontal line on a circular turntable (see Figure 1, top panel). After explaining to the child “we are going to take turns in this game,” the experimenter used a hammer to tap the left, the center, and then the right cylinder. She then placed the hammer on the turntable and spun it until the hammer was in front of the child. After three rounds of the game, a 10 s interruption period was employed. During the interruption period, the experimenter had a neutral expression and avoided making eye contact with the child. After the interruption period was complete, the child and experimenter completed three more rounds of the game. The experimenter coded for how successfully the child performed the task. The child received a point if he/she hammered the cylinders, if he/she placed the hammer on the turntable, and if he/she turned the turntable. The child received an additional half of a point if he/she hammered in the correct sequence. The child also received a half of a point if he/she handed the hammer to the experimenter instead of placing it on the turntable. The child's behavior during the interruption period was coded for whether the child was disengaged or partner oriented.

### Social motor coordination and movement tests

#### Imitation tasks

A battery of imitation tasks was developed by the experimenters to standardize the types of tasks so that they were equivalent in movement sequences, complexity, and task context. We used imitation tasks that employed similar action sequences for object-directed (body-object, object-object), body-directed (body-body), and space-directed (body-alone, face-alone) movements. When administered, children sat at a table facing the experimenter (see Figure 1, middle panel). During each task, the experimenter demonstrated the action and prompted the child to imitate by saying “It's your turn.” She then repeated the action and prompted the child to imitate two more times. During the object-object and body-object tasks, the child and experimenter each had a set of three different colored plastic cylinders positioned in front of them on the table. In the object-object task, the experimenter used a wooden hammer to tap each of the cylinders in order from left to right. In the body-object condition, the experimenter followed the same procedure, but used her pointer finger to tap each of the drums rather than using a hammer. After these two tasks, the experimenter removed the plastic cylinders from the table. During the body-body task, the experimenter used her pointer finger to tap her left shoulder, the center of her chest, and then her right shoulder. In the body alone task, the experimenter used her pointer finger to tap a point in space approximately 12 cm in front of her left shoulder, the center of her chest, and then her right shoulder. During the face alone task, the experimenter stuck out her tongue as she moved her head to the same three points in space as during the body-alone task. The quality of the child's imitation on each item of the imitation battery was coded. The child was awarded 1 point if he/she exhibited similar movement to that of the experimenter. Similar movement was defined as a clear attempt to imitate the experimenter. The child received an additional 0.5 point if he/she made three correct actions and another 0.5 point if he/she performed the three correct actions in the correct sequence. Correct actions were defined as three distinct movements toward a different location in space.

#### Social synchronization tasks

A set of synchronization tasks was developed that consisted of the same five kinds of movements as the imitation battery. After the initial demonstration of the movement, the experimenter prompted the child to perform the action with them in synchrony by saying, “Now, let's try it a few times together” so that the child and the experimenter performed the movements at the same time. The purpose of this synchronization battery was to determine how well the child coordinated their movements with the experimenter in time.

#### Motor coordination tasks

The degree of manual motor dexterity was evaluated using three different drumming tasks. For all three tasks, movement acquisition Polhemus Liberty sensors (Polhemus Corporation, Colchester, VT) were attached to the hammers used by the child to drum (see Figure 1, bottom panel). In the single hand task, a plastic cylinder was placed on the table in front of the child and he/she was given a hammer. After watching a 10 s demonstration by the experimenter, the child was prompted to drum for 15 s using his/her dominant hand. A second drum and hammer were used for the inphase (i.e., hitting the two drums at the same time with the two hammers) and antiphase (i.e., hitting the two drums in alternation with the two hammers) bimanual drumming tasks. After a 10 s demonstration of inphase drumming, the experimenter prompted the child to drum in the same manner for 15 s. The experimenter followed the same procedure for the antiphase task.

### Procedure

Each child was tested individually and the experimental session lasted approximately 45 min. The experimental protocol was piloted with two TD children (not included in the data analysis). After that, experimental sessions with ASD and TD participants were scheduled based on availability such that sessions for ASD and TD participants were interleaved. Two female experimenters carried out the experimental session. One performed the tasks with the children while the other was responsible for bringing experimental materials into the room at the appropriate time. The entire experimental session was recorded using a Mangold Multi-media workstation with four Sony Handycam camcorders. One camera focused on the child, another was focused on the experimenter, and the two other cameras offered overhead views of the table where experimenter and participant were seated. Children were randomly assigned to either perform the imitation or synchrony tasks. After a brief familiarization period in which the experimenters oriented parent and child to the experimental setup, the experimenter led the child into the testing room. The order of presentation of the experimental conditions was randomly chosen. Given the complexity of the experimental design, the order of presentation of conditions was the same for all participants.

Once in the testing room, the child was seated at a table facing the experimenter. In front of both the child and experimenter were three plastic cylinders and a wooden hammer. The child either performed the synchrony or imitation battery. Next, the experimenter initiated the pen helping task using materials that had previously been placed under the table. The, child participated in the first initiating joint attention task with a wind-up toy. To perform the motor control battery, the experimenter placed a cylinder in front of the child and placed a hammer in his/her dominant hand. Polhemus Liberty system sensors were attached to the hammers. After watching a brief demonstration by the experimenter, the child completed the single hand, in-phase and anti-phase drumming tasks. The experimenter removed the cylinders from the table and led the child through the first responding to joint attention task.

Next, the helping task with paper balls and the second initiating joint attention task using a mechanical toy were performed. Following these tasks, a second experimenter entered to demonstrate the double tube cooperation task and the double tube task cooperation task (Warneken et al., 2006) was performed. Next the turn-taking cooperation task, the theory of mind task, and the second responding to joint attention task were completed in sequence. Finally, the intentionality tasks (the dumbbell, the prong and loop, the square and post and the cylinder and beads) were performed followed by the third initiating joint attention task with a windup toy. The child was then reunited with his/her parent.

### Analyses

The cognitive social coordination measures were coded using Mangold Interact software using the behavioral codes as outlined above. The second author served as the primary coder and was not blind to the experimental conditions. The measures of motor coordination and imitation/synchrony tasks required analyses of the participants' movement. To examine motor coordination, experimenters analyzed time series data collected using the Polhemus Liberty system during the drumming tasks. Using analysis routines written in Matlab, we calculated the period and period standard deviation for the single-handed drumming task, as well as the dominant and non-dominant hands of the inphase and antiphase bimanual drumming tasks. Additionally, to evaluate the degree of coordination in the inphase and antiphase drumming tasks the relative phasing of the wrist time series was evaluated. Relative phase is an angle that measures where one rhythm is in its cycle (i.e., its phase) with respect to where another rhythm is in its cycle. If two rhythms are in identical parts of their cycles at the same time, they have a relative phase of 0° and are inphase. If two rhythms are in opposite parts of their cycles, they have a relative phase of 180° and are in antiphase. To calculate the relative phasing, an instantaneous relative phase algorithm (Pikovsky et al., 2001) was employed that calculated the relative phase angle for each sample of the time series (i.e., every 8.3 ms). The calculated relative phase time series were then analyzed by finding the frequency of occurrence of the relative phase angles in each of nine 20° relative phase regions between 0° and 180° (Schmidt and O'Brien, 1997; Richardson et al., 2005). The resultant distributions of relative phase could then be used to evaluate how well the movements were inphase or antiphase by determining whether there were concentrations of relative phase angles in the 0° or 180° regions.

We also evaluated the degree to which participants exhibited bodily coordination with the experimenter during the imitation and synchrony tasks. To do so, the experimenter used the computer program Interact by Mangold, to create separate video clips of each task in the imitation or synchrony battery. Following the methodology established by Schmidt et al. (2012), experimenters used video analyses written in Matlab to evaluate the amount of pixel change between adjacent video frames which corresponds to the amount of activity of the participant or the experimenter when the only movement in the frame is that of the participant or experimenter. The video frames were first cropped to include the movements of only one person. Then the number of pixels that changed between adjacent frames was calculated for each pair of frames to indicate the amount of whole body activity that occurred for that person at that point in time. A time series of these pixel change values was created for each participant in the interaction.

Additionally, to assess the degree of coordination during the imitation and synchrony tasks, the distributions of relative phase angles formed between the two activity time series were calculated using the procedure described above for the drumming tasks. How well the participant imitated the experimenter can be determined by ascertaining the degree of alternation in the activity time series as indicated by relative phase angles near 180°since imitation is an alternation in time of activity. We would expect less socially coordinated individuals to produce a less consistent antiphase alternation of activity and hence produce fewer phase angles near 180°. How well the participant synchronized with the experimenter can be determined by the degree of inphase synchronization as indicated by relative phase angles near 0°. We would expect less socially coordinated individuals to produce a less consistent inphase activity and hence produce fewer phase angles near 0°Adjustments for violations of sphericity were made as necessary in the statistical analyses performed.

All statistical analyses were performed in SPSS Statistics 20 (IBM). The psychological tests and motor coordination tasks were evaluated using unpaired *t*-tests. The imitation, social synchronization, and motor tasks were evaluated using frequency distributions and ANOVAS. A principal components analysis was used to evaluate the relationship between the psychological, social cognitive coordination measures, and social motor coordination measures. Intrapersonal motor control data could not be included in the PCA because adequacy criteria for performing the analysis were not satisfied as a consequence of the elimination of three subjects due to experimental error. The Kaiser-Meyer-Olkin measure of sampling adequacy was below the recommended value of 0.5, and Bartlett's test of sphericity was not significant. Perhaps, more importantly adding the antiphase drumming variable led to an un-interpretable factor structure: it added an additional factor and on which only itself and the theory of mind task loaded.

## Results

### Psychological tests

In order to evaluate overall developmental differences between children with ASD and TD children, a series of *t*-tests comparing the Developmental Profile scores were conducted. Given the small *n* in this pilot study we report both statistical significant as well as describe patterns evidence in the data. As can be seen in Table 1, the typically developing children were rated by their parents to be significantly more developmentally advanced than the autistic children on physical, adaptive, social-emotional, and cognitive aspects of behavior, in spite of the fact that the two groups were not significantly different from each other in chronological age. Only the communication behavior subscale did not reach significance. Similar *t*-tests were conducted to compare the cognitive measures of social coordination of the two groups. The cognitive behavior tasks were less successful in significantly differentiating the two groups (Table 2). In all except the intentionality task, the autistic group had lower scores, but these were not statistically significant differences. The difference between the ASD and TD groups was significantly different for the partner orientation during the interruption period of the cooperation tasks and theory of mind measures approached significance. None of the helping and cooperation measures in Table 3 significantly differentiated the two groups.

**Table 1:** **Results for developmental profile subscales**.

**Subscale**	**Means**		***T*-test results**		
	***ASD***	**Typical**	***t***	***p***	***r*^2^**
Physical	29	57	2.40	0.03^*^	0.26
Adaptive	14	43	2.50	0.02^*^	0.28
Social-emotional	5	49	4.58	<0.01^*^	0.57
Cognitive	36	67	2.45	0.03^*^	0.27
Communication	26	53	1.79	0.12^ns^	0.17

df = 16.

* p < 0.05;

ns p > 0.05.

**Table 2:** **Results for cognitive tasks**.

**Task**	**Means**		***T*-test results**		
	***ASD***	**Typical**	***t***	***p***	***r*^2^**
RJA	98.9	100	0.79	0.44^ns^	0.04
IJA	10.2	14.6	1.35	0.20^ns^	0.10
Theory of mind	1.9	2.43	1.67	0.11^ns^	0.15
Intentionality	85.6	73.3	1.38	0.21^ns^	0.10
Partner orientation	72.7	100	3.09	0.01^*^	0.37

df = 16.

* p < 0.05;

ns p > 0.05.

**Table 3:** **Results for helping and cooperation tasks**.

**Task**	**Means**		***T*-test results**		
	***ASD***	**Typical**	***t***	***p***	***r*^2^**
Helping	2.91	3.00	0.79	0.44^ns^	0.04
Double tube	3.82	3.86	0.21	0.84^ns^	0.10
Turn taking	89.9	76.5	1.49	0.15^ns^	0.15

df = 16.

ns *p > 0.05*.

### Imitation tasks

**Figure 2:**
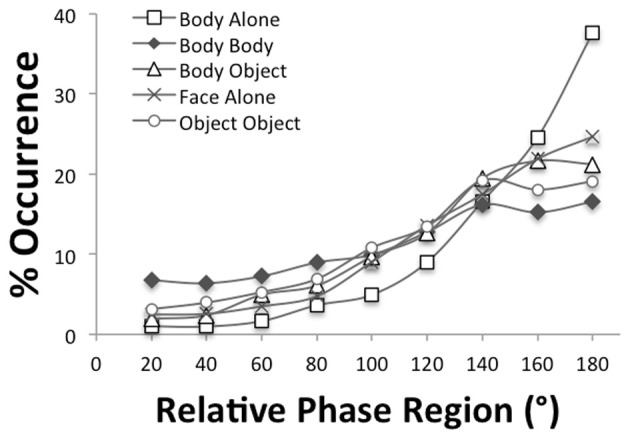
**Distributions of relative phase for the five imitation tasks**.

To evaluate the interpersonal coordination of the imitation and synchrony batteries, the relative phasing of the bodily movements was analyzed. Figure 2 displays the relative phase distributions of the five imitation tasks. The concentration of relative phase values near 180° indicates alternation of bodily movements of the participant and the experimenter as expected for imitation coordination. The plot also reveals that the body-alone task had the strongest alternation while the body-body task had the weakest alternation. A Three-Way ANOVA with between-subjects variable of group (autism, typical) and within-subjects variables of task (body-alone, body-body, body-object, face-alone, object-object) and relative phase region (0–20, 21–40, …, 161–180) verified this observation yielding a significant interaction between task and region, *F*_(11.36, 256)_ = 6.66, *p* < 0.001, η^2^_*p*_ = 0.45. No main effects were significant. A follow-up One-Way ANOVA that compares the five tasks was performed on the average of the concentrations at the relative phase regions that define alternation (i.e., the 141–160° and 161–180° regions) found that indeed body-alone had significantly greater alternation than the four other tasks (all *p* < 0.05) and that the body-body task had significantly less alternation than all but the object-object task (all *p* < 0.05). Importantly, the Three-Way ANOVA revealed no significant effects of group suggesting that both autistic and typically developing participants found these same imitation tasks equally easy or difficult to perform.

### Social synchronization tasks

**Figure 3:**
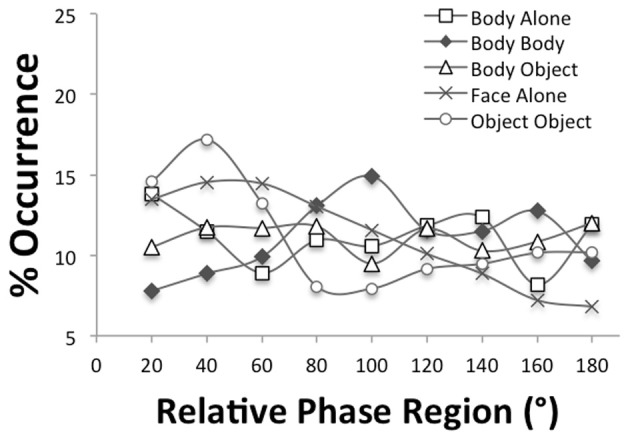
**Distributions of relative phase for the five synchronization tasks**.

Figure 3 displays the relative phase distributions of the five synchronization tasks. A concentration of relative phase values near 0° would indicate inphase synchronization. Since chance synchronization would yield a flat distribution with average values of 11.11%, the figure reveals overall low synchronization across the tasks suggesting that the synchronization task was somewhat harder to perform for the participants. In some of the tasks, such as object-object, face-alone and body alone, greater inphase coordination occurred as indicated by the higher concentration of relative phase values near 0°. A Three-Way ANOVA with between-subjects variable of group (autism, typical) and within-subjects variables of task (body-alone, body-body, body-object, face-alone, object-object) and relative phase region (0–20, 21–40, …, 161–180) revealed a significant interaction of task and region [*F*_(21.3, 127.8)_ = 2.32, *p* < 0.01, η^2^_*p*_ = 0.28] as well as of group, task and region, [*F*_(21.3, 127.8)_ = 2.05, *p* < 0.01, η^2^_*p*_ = 0.26]. No main effects were significant. A Two-Way ANOVA with variables of group and task performed on the average of the concentrations at the relative phase regions specific to inphase synchronization (i.e., the 0–20° and 21–40° regions) yielded a significant main effect of task, *F*_(3.5, 20.9)_ = 4.3, *p* < 0.05, η^2^_*p*_ = 0.42, and significant interaction of group and task, *F*_(3.5, 20.9)_ = 2.93, *p* = 0.05, η^2^_*p*_= 0.33. Follow-up tests on the main effect indicated that the object-object task had significantly more synchronization than all of the other tasks (*p* < 0.05) except for face-alone. The analysis of the interaction demonstrated that the typically developing group alone showed greater synchronization for the object-object task.

### Motor coordination tasks

**Figure 4:**
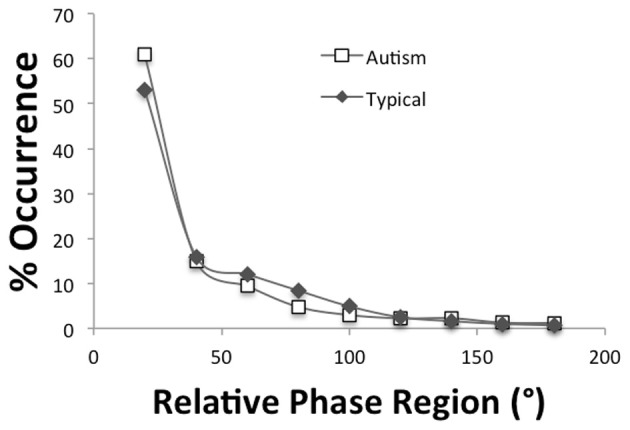
**Distributions of relative phase for inphase drumming**.

**Figure 5:**
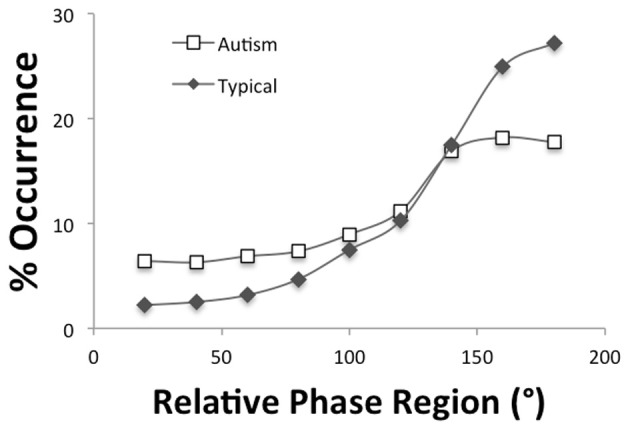
**Distributions of relative phase for antiphase drumming**.

The motor coordination data of three participants were lost due to experimenter error, thereby, reducing the overall *n* to 15 participants, 8 ASD, and 7 TD. Independent *t*-tests were performed to determined if the tempo (e.g., the frequency of the movement) and tempo variability differed (using period and period SD measures, respectively) between the autism and the typically developing groups for the single hand as well as the bimanual inphase and antiphase drumming. As can be seen in Table 4, the autism group tended to be slower in tempo as well as more variable although it is only in the more difficult antiphase drumming that significant group differences and larger effect sizes appear. A mixed design ANOVA with a between-subjects variable of group (autism, typical) and within-subjects variable of relative phase region (0–20, 21–40, …, 161–180) performed on the distributions of relative phase values calculated for inphase drumming revealed a main effect of relative phase region [*F*_(1.68, 21.8)_ = 119.8, *p* < 0.001, η^2^_*p*_= 0.90] but no effects of group. As Figure 4 demonstrates, large concentration of relative phase values were observed near 0° phase indicating that the drumming of the two hands occurred synchronously. A similar ANOVA performed on the distribution of relative phase values calculated for antiphase drumming revealed a main effect of relative phase region [*F*_(1.6, 20.9)_ = 22.5, *p* < 0.001, η^2^_*p*_= 0.63] but no significant interaction between group and region [*F*_(1.6, 20.9)_ = 2.45, *p* = 0.12, η^2^_*p*_= 0.16]. As Figure 5 displays and follow-up tests revealed, the autism group produced during antiphase drumming had slightly higher concentrations in the 0–20° and 21–40° inphase relative phase regions (*p* = 0.10 and 0.07, respectively) and slightly lower concentrations in the 161–180° antiphase relative phase regions (*p* = 0.10).

**Table 4:** **Results for drumming tempo and variability**.

**Task**	**Means**		***T*-test results**		
	***ASD***	**Typical**	***t***	***p***	***r*^2^**
**SINGLE HAND**					
Period	0.71	0.35	0.98	0.36^ns^	0.07
Period SD	0.42	0.04	1.10	0.31^ns^	0.09
**INPHASE BIMANUAL**					
Dominant period	0.76	0.77	0.21	0.83^ns^	0.01
Dominant period SD	0.11	0.08	0.62	0.55^ns^	0.03
Non-dominant period	0.75	0.76	0.19	0.85^ns^	0.01
Non-dominant period SD	0.08	0.07	0.32	0.75^ns^	0.01
**ANTIPHASE BIMANUAL**					
Dominant period	0.74	0.66	2.49	0.03^*^	0.33
Dominant period SD	0.15	0.13	0.51	0.62^ns^	0.02
Non-dominant period	0.77	0.65	3.14	<0.01^*^	0.43
Non-dominant period SD	0.19	0.12	1.90	0.08^ns^	0.22

* p < 0.05;

ns *p > 0.05*.

### Relationship between motor coordination and psychological tasks

In order to determine the relationship between the various psychological tests and cognitive measures of social coordination (Developmental Profile III, RJA, IJA, theory of mind, intentionality, partner orientation during cooperation tasks, and cooperation the measures of social motor coordination), a principal components analysis was performed. Principle components analysis is used to determine whether there are latent factors or components underlying the correlations between variables measuring different aspects of a phenomenon. For our purposes we are interested in whether all the psychological tests are measuring the same or different aspects that differentiate autism from typically developing children as well as whether these traditional measures of autistic competence relate to the embodied measures of social motor coordination.

The psychological tests that had the largest effect size in differentiating the two groups were chosen for this analysis. These included the social-emotion and adaptive subscales from Development Profile as well as five cognitive and cooperation tests: initiating joint attention, theory of mind, partner orientation during the interruption period, intentionality and turn taking. As for an index of social motor coordination, the percentages that the participants were in the dominant regions for imitation or synchronization (i.e., either the 141–160° and 161–180° regions for imitation or the 0–20° and 21–40° regions for synchronization depending on which test they received) were used. The performed principal components analysis satisfied several adequacy criteria. First, all items correlated at least 0.3 with at least one other item, suggesting reasonable factorability. Second, the Kaiser-Meyer-Olkin measure of sampling adequacy was above the recommended value of 0.5, and Bartlett's test of sphericity was significant [χ^2^(28) = 43.3, *p* < 0.05]. Additionally, the communalities were all above 0.5 confirming that each item shared some common variance with other items.

A principal components analysis using varimax (orthogonal) rotation found that the three factors explained 73% of the variance. The loadings less than 0.40 were excluded. The results of this solution are shown in Table 5. A replication of the analysis using an oblimin (oblique) solution showed little difference. Four items, the social-emotional subscale, initiating joint attention, partner orientation during the interruption periods and the adaptive subscale, loaded onto factor 1 that explained 32% of the variance. This factor seems to be indexing social attention aspects of the interactions between the participant and the experimenter. The orientation of the participant to the experimenter during the interruption periods and the initiating joint attention obviously test this and arguably the mothers' judgment of the child's social-emotional and adaptive behavior on the Developmental profile is representing the kind of mental connectedness they perceived the child to have. Three items, theory of mind, turn taking and social motor coordination, loaded onto factor 2 that explained 24% of the variance. This factor seems to be indexing social knowledge that the participant demonstrated. The theory of mind task measures how well the child can see the world from another's point of view and this kind of knowledge is necessary for performing cooperative acts like turn taking with another person. Interestingly, the social motor coordination measure loaded on the social knowledge factor rather than the social attention factor. The final factor was comprised of two items, the intentionality test and social motor coordination, and explained an additional 17% of the variance. The intentionality test was designed to measure whether a child understood the goal of an action that another person was performing. However, in that the test consists of replicating failed actions of another, it contains a large social motor component. Consequently, it is not surprising to see social motor coordination related to it. What is surprising but not unprecedented is that the intentionality test defined a separate factor and did not load on the factor 2 which defined the social knowledge of perspective taking.

**Table 5:** **Results of the principal components analysis**.

**Item**	**Factor 1**	**Factor 2**	**Factor 3**
Social-emotional	0.85		
IJA	0.77		
Theory of mind		0.74	
Turn taking		0.92	
Partner orientation	0.76		
Intentionality			0.87
Social motor coordination		0.50	0.64
Adaptive	0.77		

## Discussion

Parents rated the children with ASD lower on all the parental-report rating scales (physical, adaptive, social-emotion, and cognitive) except communication, but children with ASD were not significantly different from TD children on most of the social cognitive tasks (IJA, RJA, theory of mind, behavioral reenactment intentionality). These results suggest that, as predicted, overall task success measures of the social cognitive tasks may not be the most sensitive or effective way to differentiate children with ASD and TD children. Alternatively, ceiling effects on some of these measures may have made it difficult to distinguish between the groups. Future research should explore whether other measures could be used to avoid such ceiling effects.

The lack of an ASD deficit on both the theory of mind task and the behavioral reenactment intentionality task suggest that the children with ASD may have the ability to understand intentions. The high verbal ability of our participants likely contributed to the success on the theory of mind task. However, even though the ASD children were not significantly different from the TD children on theory of mind scores, the effect size of this test indicates that it corresponds to a medium effect (Cohen, 1988) suggesting that the lack of significance observed was a Type II error. Our findings on the behavioral reenactment intentionality task, however, are consistent with past research that also found that children with ASD were equivalent to (if not better than) TD children on behavioral reenactment tasks (Aldridge et al., 2000; Carpenter et al., 2001; Colombi et al., 2009). Although this task has been thought to indicate a participant's understanding of another's intentions, Colombi et al. (2009) argue that understanding intentions may not be the same as *sharing* intentions, which may be at the heart of the ASD social disorder. Moreover, Carpenter et al. (2001) report that children with ASD did not complete the reenactment tasks using the same *style* as the experimenter did. This suggests that the manner in which the exchanges unfold over time may be more important than the task outcome itself. In future research, we plan to analyze the structure of the movements during the behavioral reenactment tasks to explore whether the movement execution of the reenactment tasks differentiates the two groups. Furthermore, Huang et al. (2002) have questioned whether Meltzoff's behavioral reenactment tasks actually demonstrate intentional attribution. They argue instead that stimulus enhancement, emulation learning, and object affordances may be a more parsimonious explanation of the behavioral reenactment results. Our principal components analysis in which the theory of mind task and the behavioral reenactment intentionality task loaded on separate factors lends some credibility to the argument that these two measures may not be measuring the same underlying construct. More research is needed examine this possibility.

Partner orientation during the interruption phase of cooperation tasks did significantly differentiate the two groups. Children with ASD were significantly worse than TD children on the partner orientation tasks. Colombi et al. (2009) and Liebal et al. (2008) report similar findings and take this as evidence that children with ASD have trouble sharing intentions even if they are able to understand them. In our principal components analysis the partner orientation loaded onto our “social attention” factor along with initiating joint attention and social-emotional and adaptive scores. It is possible that the sharing of intention is related to disruptions in lower-level perceptual or attentional processes. For example, the complex, time-dependent nature of social exchanges requires that children shift attention between both the instrumental task and the partner they are interacting with. Since research has demonstrated that children with ASD have profound atypical persistence in focus and resistance to distraction (Gernsbacher et al., 2008) and during naturalistic social interactions visually fixate on mouths and objects rather than eyes (Klin et al., 2002), the lack of social sharing may be related to the problems in attending to the relevant social information. Similarly, Sasson et al. (2007) reported that individuals with autism have deficiencies in basic social perception and orienting to social stimuli.

To evaluate the imitation tasks, we used dynamical measures that evaluate *how* the tasks were performed. We found that both the ASD group and the TD group accomplished the tasks and demonstrated coordinated alternation of movements. Our results are consistent with others who also found that individuals with autism were equivalent to those without autism in imitation performance (e.g., Hamilton et al., 2007). However, we did find some evidence for an ASD deficit in simultaneous movement synchronization (i.e., in the object–object synchrony task). Overall, these tasks were more difficult for both groups because they require a more fine degree of temporal coordination and consequently, perhaps it is not so surprising that group differences are revealed here. The subtleness of the group differences revealed could be due to our calculating relative phase using whole-body movements. In future research, we plan to conduct a more fine-grained analysis of the hand movements employed during the imitation and synchrony tasks to be able to compare the findings to the whole-body movements and determine whether a similar pattern of results emerges.

It is quite new to look at imitative motor movements in terms of a relative phase measure. There is some precedence for this in Wilson and Wilson's (2005) coupled oscillator modeling of turn-taking behavior in speech. To understand the utility of using relative phase for imitative motor movements, one must remember that we are measuring activity and one should expect to see an alternation of repeated activity in imitation. The relative phasing of activity can be understood as quantifying a continuum of perfectly simultaneous repeated activity (0°) to perfectly alternating repeated activity (180°). Any variability in the alternating, turn-taking activity during imitation will be resolved in the distribution of relative phasing as values away from 180°. Unlike cross-correlation measures, the distribution of relative phase values has the utility of portraying the patterning of these deviations of perfect synchrony/alternation. Consequently, we would expect less socially coordinated individuals to have less consistent time delays, and hence, flatter distribution of relative phase.

Our finding that we did not see deficits in the joint attention behavior of children with ASD is a bit curious since it is widely reported in the literature that children with ASD perform poorly on joint attention tasks (Sigman and Ungerer, 1984; Sigman et al., 1986; Baron-Cohen, 1989; Sigman and Mundy, 1989; Kasari et al., 1990; Charman et al., 1997; Leekam et al., 1997; Bono et al., 2004), although a dissociation between IJA and RJA has been reported (Mundy et al., 1994, 1995). One possible reason for this discrepancy is that our sample size was just too small to see significant effects for IJA because the effect size was at the low end of a medium effect size (Cohen, 1988). Alternatively, research suggests there is a relationship between joint attention and language ability (Tomasello and Todd, 1983) as well as conversation skill (Farrant et al., 2011). Relatedly, even in typical development there is an association between joint attention and social competence, with individual differences predictive of social outcomes (Vaughan Van Hecke et al., 2007). Since our ASD sample was high-functioning it is likely that our participants were at the high end of the joint attention skill spectrum. As Mundy (2009) points out, blanket statements about the social behaviors of children with autism are problematic because *some* children with autism do display some level of IJA and RJA. A larger and more diverse sample is necessary to explore this issue in more depth.

Dowd et al. (2010) argue that motor function is important because interpersonal interactions and communication rely on motor function for execution (e.g., both speech and gesture involve motor asks) and because social deficits and motor deficits may share similar neural circuits. Similarly, Gernsbacher et al. (2008) proposed that the difficulties that children with ASD have in initiating joint attention may result not from a lack of understanding of intentionality but may be due to a core deficit in motor control. However, it is worth noting that performing motor tasks depend not only on motor skill but also the ability to attend to and imitate another person thus making it difficult to determine which is the core deficit. We would argue that motor tasks tend to involve more stereotypical movements that, in the context of our experiment at least, have already been learned while imitative sequences tend to involve a novel pattern of movements specific to the task context. While this is an issue that future research certainly needs to address, taking careful measures of these variables to be able to evaluate relationships between them is an important first step.

Our dynamical measures of motor control during the drumming task found significant group differences only for the bimanual anti-phase drumming condition. Isenhower et al. (2012) found that children with autism exhibited less in-phase and anti-phase coordination than typically developing children on a similar drumming task. They suggest that such motor control deficits impair the development of social coordination because the same coordinative processes underlying bimanual interlimb coordination have also been found to constrain the rhythmic coordination between an individual and either an environmental rhythm (Schmidt et al., 2007) or another individual (Schmidt and O'Brien, 1997). Hamilton et al. (2007) found that children with ASD were equivalent in terms of motor planning, but they did report an association between verbal ability and motor planning. Since our participants had high verbal abilities this may explain the weaker differences in motor ability that we observed. In future research we plan to investigate motor control across the autism spectrum to determine the effect of such relationships.

Although we were not able to evaluate the relation between cognitive social coordination measures and intrapersonal motor control (measured in the drumming task) due to data loss as a consequence of equipment malfunction, we did find that understanding of intentionality (in theory of mind, behavioral reenactment intentionality, and partner orientation) did load with our social (interpersonal) motor control measure. This suggests that disruptions in executing movements in a social situation may be important for understanding the social deficits in ASD and points to the promise of this research methodology. Our principal components analysis suggests that different factors measure unique aspects of social coordination, lending credence to the idea that social competence may not be a unitary construct. As mentioned, we identified three separate factors—social attention accounting for 32% of the variability, social knowledge (24%), and social action (17%). Initiating joint attention and partner orientation during the interruption periods (along with the social-emotional and adaptive parent report subscales) all loaded onto the same factor. These measures seem to be measuring the lower-level perceptual and attentional dimensions of social competence and could be referred to as Social Attention. Interestingly, however, these lower-level, perceptually-based measures of social coordination did not load on the same factor as higher-level, more conceptually-based measures of social competence, which we are calling Social Knowledge. This raises the possibility that these may be separate and distinct dimensions of social competence with non-shared underlying mechanisms. This is consistent with previous research that found dissociations between lower-level and higher-level social cognition when comparing individuals with autism and schizophrenia (Sasson et al., 2007, 2011). In particular, they found that those with autism perform poorly on both basic social perception and higher-level social cognitive skill while those with schizophrenia do not demonstrate deficits on basic social perception but are similar to those with autism in higher-level social cognitive skills. At any event, the more perceptually-based measures of social competence did not load on the same factor as social motor coordination, what we refer to as Social Action, as we predicted. In future research we plan to explore whether perceptual-based measures of social coordination are related to more basic intrapersonal motor control measures while higher-level social cognitive skill is related to social motor measures. The utility of these three factors in diagnosing ASD-specific deficits in social competence is an interesting avenue for future research.

We did find, however, that both theory of mind and behavioral reenactment intentionality were related to social-motor coordination, although they loaded on separate factors. This finding raises two important issues. First, as mentioned previously, it lends credence to arguments that the behavioral reenactment tasks may not be measuring the same aspects of intentionality as the theory of mind tasks (Huang et al., 2002). In addition, it suggests that our motor movements in social interactions are related to the intentional processes underlying them. In short, the mind is embodied in our social interactions with others. This supports our prediction that social motor coordination is an important pathway for understanding social coordination and may provide important insights into understanding the social deficits in ASD.

In conclusion, our findings suggest that focusing on *whether* children can accomplish a task may not adequately capture the nature of the social deficits in ASD. The experimental methodology that we have outlined here–standardizing tasks and movement sequences across a variety of social cognitive and social motor tasks and measuring the dynamic unfolding of social motor behavior across a spectrum of social skills—holds much promise for advancing our understanding of deficit-specific processes and perhaps disorder-specific deficits in ASD. While the small *n* in this study does warrant caution in drawing conclusions, our findings do suggest that social motor coordination is an important avenue for continued research to understand whether social coordination is a unitary construct and identify the deficit-specific underlying mechanisms in ASD. By including such diverse measures of social coordination, this method holds much promise for bridging the gap in what we understand about ASD social deficits from empirical research, clinical research and observation, and naturalistic social interactions.

### Conflict of interest statement

The authors declare that the research was conducted in the absence of any commercial or financial relationships that could be construed as a potential conflict of interest.

## References

[B1] Aldridge M. A. Stone K. R. Sweeney M. H. Bower T. G. R. (2000). Preverbal children with autism understand the intentions of others. Dev. Sci. 3, 294–301

[B2] Alpern G. D. (2007). Developmental Profile 3 (DP-3). Los Angeles, CA: Western Psychological Services

[B3] American Psychiatric Association. (2000). Diagnostic and Statistical Manual of Mental Disorders, 4th Edn-TR Washington, DC: Task Force

[B4] Baron-Cohen S. (1989). Perceptual role taking and protodeclarative pointing in autism. Br. J. Dev. Psychol. 7, 113–127

[B5] Baron-Cohen S. (1995). Mind-blindness: An Essay on Autism and Theory of Mind. Cambridge, MA: MIT Press/Bradford Books

[B6] Baron-Cohen S. Swettenham J. (1997). Theory of mind in autism: Its relationship to executive function and central coherence, in Handbook of Autism and Pervasive Developmental Disorders, eds Cohen D. J. Volkmar F. R. (New York, NY: Wiley), 880–893

[B7] Baron-Cohen S. Leslie A. M. Frith U. (1985). Does the autistic child have a “theory of mind?” Cognition 21, 37–46 10.1016/0010-0277(85)90022-8 2934210

[B8] Bauminger N. (2002). The facilitation of social-emotional understanding and social interaction in high-functioning children with autism: intervention outcomes. J. Autism Dev. Disord. 32, 283–298 12199133 10.1023/a:1016378718278

[B9] Bernieri F. J. Davis J. M. Rosenthal R. Knee C. R. (1994). Interactional synchrony and rapport: measuring synchrony in displays devoid of sound and facial affect. Pers. Soc. Psychol. Bull. 30, 303–311

[B10] Bono M. A. Daley T. Sigman M. (2004). Relations among joint attention, amount of intervention and language gain in autism. J. Autism Dev. Disord. 34, 495–505 15628604 10.1007/s10803-004-2545-x

[B11] Carpenter M. Call J. Tomasello M. (2002). A new false belief test for 36-month-olds. Br. J. Dev. Psychol. 20, 393–420

[B12] Carpenter M. Pennington B. Rogers S. (2001). Understanding of others' intentions in children with autism. J. Autism Dev. Disord. 31, 589–599 11814270 10.1023/a:1013251112392

[B13] Charman T. Swettenham J. Baron-Cohen S. Cox A. Baird G. Drew A. (1997). Infants with autism: an investigation of empathy, pretend play, joint attention, and imitation. Dev. Psychol. 33, 781–789 10.1037/0012-1649.33.5.781 9300211

[B14] Chartrand T. Bargh J. (1999). The chameleon effect: the perception-behavior link and social interaction. J. Pers. Soc. Psychol. 76, 893–910 10.1037/0022-3514.76.6.893 10402679

[B15] Colombi C. Liebal K. Tomasello M. Young G. Warneken F. Rogers S. (2009). Examining the correlates of cooperation in autism: imitation, joint attention, and understanding intentions. Autism 13, 143–163 10.1177/1362361308098514 19261685

[B16] Cohen J. (1988). Statistical Power Analysis for the Behavioral Sciences, 2nd Edn Hillsdale, NJ: Lawrence Earlbaum Associates

[B17] Dale R. Spivey M. J. (2006). Unraveling the dyad: using recurrence analysis to explore patterns of syntactic coordination between children and caregivers in conversation. Lang. Learn. 56, 391–430

[B18] Dowd A. M. Rinehart N. J. McGinley J. (2010). Motor function in children with autism: why is this relevant to psychologists? Clin. Psychol. 14, 90–96

[B19] Fan Y. Decety J. Yang C. Liu J. Cheng Y. (2010). Unbroken mirror neurons in autism spectrum disorders. J. Child Psychol. Psychiatry 51, 981–988 10.1111/j.1469-7610.2010.02269.x 20524939

[B20] Farrant B. M. Maybery M. T. Fletcher J. (2011). Socio-emotional engagement, joint attention, imitation, and conversational skill: analysis in typical development and specific language impairments. First Lang. 31, 23–46

[B21] Feldman R. (2007). Parent–infant synchrony and the construction of shared timing; physiological precursors, developmental outcomes, and risk conditions. J. Child Psychol. Psychiatry 48, 329–354 10.1111/j.1469-7610.2006.01701.x 17355401

[B22] Gallese V. (2006). Intentional attunement: a neurophysiological perspective on social cognition and its disruption in autism. Cogn. Brain Res. 1079, 15–24 16680812 10.1016/j.brainres.2006.01.054

[B23] Gernsbacher M. A. Stevenson J. L. Khandakar S. Goldsmith H. H. (2008). Why does joint attention look atypical in autism? Child Dev. Perspect. 2, 38–45 10.1111/j.1750-8606.2008.00039.x PMC4266470 25520747

[B24] Gowen E. Stanley J. Miall C. (2008). Movement interference in autism-spectrum disorder. Neuropsychologia 46, 1060–1068 10.1016/j.neuropsychologia.2007.11.004 18096192 PMC6010145

[B25] Gratier M. Apter-Danon G. (2008). The musicality of belonging: repetition and variation in mother-infant vocal interaction, in Communicative Musicality: Narratives of Expressive Gesture and Being Human, eds Malloch S. Trevarthen C. (Oxford: Oxford University Press), 301–327

[B26] Hamilton A. F. Brindley R. M. Frith U. (2007). Imitation and action understanding in autistic spectrum disorders: how valid is the hypothesis of a deficit in the mirror neuron system? Neuropsychologia 45, 1859–1868 10.1016/j.neuropsychologia.2006.11.022 17234218

[B27] Happe F. Frith U. (2006). The weak coherence account: detail-focused cognitive style in autism spectrum disorders. J. Autism Dev. Disord. 36, 5–25 10.1007/s10803-005-0039-0 16450045

[B28] Howlin P. Goode S. Hutton J. Rutter M. (2004). Adult outcome for children with autism. J. Child Psychol. Psychiatry 45, 212–229 10.1111/j.1469-7610.2004.00215.x 14982237

[B29] Huang C. Heyes C. Charman T. (2002). Infants' behavioral reenactment of “failed attempts”: exploring the roles of emulation learning, stimulus enhancement, and understanding of intentions. Dev. Psychol. 38, 840–855 10.1037/0012-1649.38.5.840 12220059

[B31] Isenhower R. W. Marsh K. L. Richardson M. J. Helt M. Schmidt R. C. Fein D. (2012). Rhythmic bimanual coordination is impaired in children with autism spectrum disorder. Res. Autism Spectr. Disord. 6, 25–31

[B32] Julien D. Brault M. Chartrand E. Begin J. (2000). Immediacy behaviours and synchrony in satisfied and dissatisfied couples. Can. J. Behav. Sci. 32, 84–90

[B33] Kasari C. Sigman M. Mundy P. Yirmiya N. (1990). Affective sharing in the context of joint attention interactions of normal, autistic, and mentally retarded children. J. Autism Dev. Disord. 20, 87–99 2139025 10.1007/BF02206859

[B34] Klin A. Jones W. Schultz R. Volkmar F. Cohen D. (2002). Visual fixation patterns during viewing of naturalistic social situations as predictors of social competence in individuals with autism. Arch. Gen. Psychiatry 59, 809–816 10.1001/archpsyc.59.9.809 12215080

[B35] Knoblich G. Sebanz N. (2006). The social nature of perception and action. Curr. Dir. Psychol. Sci. 15, 99–104

[B36] Lakin J. Chartrand T. L. (2003). Using nonconscious behavioral mimicry to create affiliation and rapport. Psychol. Sci. 14, 334–339 10.1111/1467-9280.14481 12807406

[B37] Leekam S. Baron-Cohen S. Perrett D. Milders M. Brown S. (1997). Eye-direction detection: a dissociation between geometric and joint attention skills in autism. Br. J. Dev. Psychol. 15, 77–95

[B38] Liebal K. Colombi C. Rogers S. Warneken F. Tomasello M. (2008). Helping and cooperation in children with autism. J. Autism Dev. Disord. 38, 224–238 10.1007/s10803-007-0381-5 17694374 PMC2758368

[B39] Meltzoff A. N. (1995). Understanding the intentions of others: re-enactment of intended acts by 18-month-old children. Dev. Psychol. 31, 838–850 10.1007/s10339-012-0518-0 25147406 PMC4137788

[B41a] Meltzoff A. N. (2005). Imitation and other minds: the “Like Me” hypothesis, in Perspectives on Imitation: From neuroscience to Social Science, Volume 2: Imitation, Human Development, and Culture, eds Hurley S. Chater N. (Cambridge, MA: MIT Press), 55–77

[B43] Miles L. K. Nind L. K. Macrae C. N. (2009). The rhythm of rapport: interpersonal synchrony and social perception. J. Exp. Soc. Psychol. 45, 585–589

[B44] Mundy P. (2009). Lessons learned from autism: an information-processing model of joint attention and social cognition, in Minnesota Symposium on Child Psychology: Meeting the Challenge of Translational Research in Child Psychology, Vol. 35, eds Cicchetti D. Gunnar M. R. (Hoboken, NJ: John Wiley and Sons), 59–113

[B45] Mundy P. Delgado C. Block J. Venezia M. Hogan A. Seibert J. (2003). A Manual for the Abridged Early Social Communication Scales (ESCS). Coral Cables, FL: University of Miami

[B46] Mundy P. Kasari C. Sigman M. Ruskin E. (1995). Nonverbal communication and early language Down syndrome and in normally developing children. J. Speech Hear. Res. 38, 157–167 7537345 10.1044/jshr.3801.157

[B47] Mundy P. Newell L. (2007). Attention, joint attention, and social cognition. Curr. Dir. Psychol. Sci. 16, 269–274 10.1111/j.1467-8721.2007.00518.x 19343102 PMC2663908

[B48] Mundy P. Sigman C. Kasari C. (1994). Joint attention, developmental level and symptom presentation in autism. Dev. Psychopathol. 6, 389–402

[B49] Oberman L. M. Ramachandran V. S. (2007). The simulating social mind: the role of the mirror neuron system and simulation in the social and communicative deficits of autism spectrum disorders. Psychol. Bull. 133, 310–327 10.1037/0033-2909.133.2.310 17338602

[B50] Onishi K. H. Baillargeon R. (2005). Do 15-month-old infants understand false beliefs? Science 308, 255–258 10.1126/science.1107621 15821091 PMC3357322

[B51] Paxton A. Dale R. (in press). Frame-differencing methods for measuring bodily synchrony in conversation. Behav. Res. Methods 10.3758/s13428-012-0249-2 23055158

[B51a] Piaget J. (1951/1967). Play, Dreams and Imitation. London: Routledge

[B52] Pikovsky A. Rosenblum M. Kurths J. (2001). Synchronization: A Universal Concept in Nonlinear Sciences. New York, NY: Cambridge University Press

[B53] Ramseyer F. Tschachter W. (2011). Nonverbal synchrony in psychotherapy: coordinated body movement reflects relationship quality and outcome. J. Consult. Clin. Psychol. 79, 284–295 10.1037/a0023419 21639608

[B54] Reed T. (1994). Performance of autistic and control participants on three cognitive perspective taking tasks. J. Autism Dev. Disord. 24, 53–66 8188574 10.1007/BF02172212

[B55] Richardson M. J. Marsh K. L. Schmidt R. C. (2005). Effects of visual and verbal interaction on unintentional interpersonal coordination. J. Exp. Psychol. Hum. Percept. Perform. 31, 62–79 10.1037/0096-1523.31.1.62 15709863

[B56] Richardson M. J. Marsh K. L. Schmidt R. C. (2010). Challenging egocentric notions of perceiving, acting, and knowing, in The Mind in Context, eds Barrett L. F. Mesquita B. Smith E. (New York, NY: Guilford), 307–333

[B57] Rizzolatti G. Fabbri-Destro M. (2010). Mirror neurons: from discovery to autism. Exp. Brain Res. 200, 223–237 10.1007/s00221-009-2002-3 19760408

[B58] Rogers S. J. Pennington B. F. (1991). A theoretical approach to the deficits in infantile autism. Dev. Psychopathol. 3, 137–162

[B59] Rogers S. Hepburn S. Stackhouse T. Wehner E. (2003). Imitation performance in toddlers with autism and those with other developmental disorders. J. Child Psychol. 44, 763–781 10.1111/1469-7610.00162 12831120

[B60] Sasson N. J. Pinkham A. E. Carpenter K. L. H. Belger A. (2011). The benefit of directly comparing autism and schizophrenia for revealing mechanisms of social cognitive impairment. J. Neurodev. Disord. 3, 87–100 10.1007/s11689-010-9068-x 21484194 PMC3188289

[B61] Sasson N. Tsuchiya N. Hurley R. Couture S. M. Penn D. L. Adolphs R. (2007). Orienting to social stimuli differentiates social cognitive impairment in autism and schizophrenia. Neuropsychologia 45, 2580–2588 10.1016/j.neuropsychologia.2007.03.009 17459428 PMC2128257

[B62] Schmidt R. C. Richardson M. J. (2008). Dynamics of interpersonal coordination, in Coordination: Neural, Behavioral and Social Dynamics, eds Fuchs A. Jirsa V. (Heidelberg: Springer-Verlag), 281–308

[B63] Schmidt R. C. Fitzpatrick P. Caron R. Mergeche J. (2011). Understanding social motor coordination. Hum. Mov. Sci. 30, 834–845 10.1016/j.humov.2010.05.014 20817320

[B64] Schmidt R. C. Morr S. Fitzpatrick P. A. Richardson M. J. (2012). Measuring the dynamics of interactional synchrony. J. Nonverbal Behav. 36, 263–279

[B65] Schmidt R. C. Richardson M. J. Arsenault C. A. Galantucci B. (2007). Visual tracking and entrainment to an environmental rhythm. J. Exp. Psychol. Hum. Percept. Perform. 33, 860–870 10.1037/0096-1523.33.4.860 17683233

[B66] Schmidt R. O'Brien B. (1997). Evaluating the dynamics of unintended interpersonal coordination. Ecol. Psychol. 9, 189–206

[B67] Seger C. Smith E. R. (2009). The effect of synchrony with a computerized avatar on implicit prejudice, in Poster Presented at Society for Social and Personality Psychology (SPSP), February 2009 (Tampa, FL).

[B68] Semin G. R. (2007). Grounding communication: synchrony, in Social Psychology: Handbook of basic principles, 2nd Edn, eds Kruglanski A. Higgins E. T. (New York, NY: Guilford Publications), 630–649

[B69] Semin G. R. Cacioppo J. T. (2008). Grounding social cognition: synchronization, coordination, and co-regulation, in Embodied Grounding: Social, Cognitive, Affective, and Neuroscientific Approaches, eds Semin G. R. Smith E. R. (New York, NY: Cambridge University Press), 119–147

[B70] Semin G. R. Smith E. R. (2008). Embodied Grounding: Social, Cognitive, Affective, and Neuroscientific Approaches. New York, NY: Cambridge University Press

[B71] Shockley K. Richardson D. C. Dale R. (2009). Conversation and coordinative structures. Topics Cogn. Sci. 1, 305–319 10.1111/j.1756-8765.2009.01021.x 25164935

[B72] Sigman M. Mundy P. (1989). Social attachments in autistic children. J. Am. Acad. Child Adolesc. Psychiatry 28, 74–81 10.1097/00004583-198901000-00014 2464573

[B73] Sigman M. Mundy P. Sherman T. Ungerer J. (1986). Social interactions of autistic, mentally retarded, and normal children and their caregivers. J. Child Psychol. Psychiatry 27, 647–656 3771681 10.1111/j.1469-7610.1986.tb00189.x

[B74] Sigman M. Ungerer J. A. (1984). Attachment behaviors in autistic children. J. Autism Dev. Disord. 14, 231–244 6207163 10.1007/BF02409576

[B75] Smith E. R. (2008). Social relationships and groups: new insights on embodied and distributed cognition. Cogn. Syst. Res. 9, 24–32

[B76] Tomasello M. (1999). The Cultural Origins of Human Cognition. Cambridge, MA: Harvard University Press

[B77] Tomasello M. Todd J. (1983). Joint attention and lexical acquisition style. First Lang. 4, 197–211

[B77a] Trevarthen C. (1998). The concept and foundations of infant intersubjectvitity, in Intersubjective Communication and Emotion in Early Ontogeny, ed Braten S. (Cambridge: Cambridge University Press), 15–46

[B78] Trevarthen C. Daniel S. (2005). Disorganized rhythm and synchrony: early signs of autism and Rett syndrome. Brain Dev. 27, S25–S34 10.1016/j.braindev.2005.03.016 16182487

[B79] Varlet M. Marin L. Raffard S. Schmidt R. C. Capdevielle D. Boulenger J. P. (2012). Impairments of social motor coordination in schizophrenia. PLoS ONE 7:e29772 10.1371/journal.pone.0029772 22272247 PMC3260163

[B80] Vaughan Van Hecke A. Mundy P. C. Acra C. F. Block J. J. Delgado C. E. Parlade M. V. (2007). Infant joint attention, temperament, and social competence in preschool children. Child Dev. 78, 53–69 10.1111/j.1467-8624.2007.00985.x 17328693 PMC2662688

[B81] Warneken F. Chen F. Tomasello M. (2006). Cooperative activities in young children and chimpanzees. Child Dev. 77, 640–663 10.1111/j.1467-8624.2006.00895.x 16686793

[B81a] Warreyn P. Roeyers H. Oelbrandt T. de Groote I. (2005). What are you looking at? Joint attention and visual perspective taking in young children with autism spectrum disorder. J. Dev. Phys. Disabil. 17, 55–73

[B82] Wellman H. M. Cross D. Watson J. (2001). Meta-analysis of theory-of-mind development. The truth about false belief. Child Dev. 72, 655–684 10.1111/1467-8624.00304 11405571

[B83] Williams J. H. G. Whiten A. Suddendort T. Perrett D. I. (2001). Imitation, mirror neurons and autism. Neurosci. Biobehav. Rev. 25, 287–295 10.1016/S0149-7634(01)00014-8 11445135

[B84] Wilson M. Wilson T. P. (2005). An oscillator model of the timing of turn-taking. Psychon. Bull. Rev. 12, 957–968 16615316 10.3758/bf03206432

[B85] Woodward A. L. (1998). Infants selectively encode the goal object of an actor's reach. Cognition 69, 1–34 10.1016/S0010-0277(98)00058-4 9871370

[B86] Yirmiya N. Gamliel I. Pilowsky T. Feldman R. Baron-Cohen S. Sigman M. (2006). The development of siblings of children with autism at 4 and 14 months: social engagement, communication, and cognition. J. Child Psychol. Psychiatry 47, 511–523 10.1111/j.1469-7610.2005.01528.x 16671934

